# Improvements in brain mechanisms associated with the aberrant salience hypothesis of psychosis following Avatar therapy for refractory auditory verbal hallucinations

**DOI:** 10.1192/j.eurpsy.2025.892

**Published:** 2025-08-26

**Authors:** A. Fortier, A. Dumais, S. Potvin

**Affiliations:** 1 Département de psychiatrie et d’addictologie, Université de Montréal; 2 Centre de Recherche de l’Institut Universitaire en Santé Mentale de Montréal; 3 Institut National de Psychiatrie Légale Philippe-Pinel, Montréal, Canada

## Abstract

**Introduction:**

Nearly 40% of individuals with schizophrenia are resistant to medication. Treatment-resistant schizophrenia leads to increased health-risk behaviors and reduced quality of life. Avatar therapy *via* virtual reality seems to be a very promising solution for individuals with treatment-resistant schizophrenia. This therapy targets refractory auditory verbal hallucinations, one of the core positive symptoms of schizophrenia. This symptom is thought to reflect a direct experience of the aberrant salience of internal representations according to the aberrant salience hypothesis of psychosis. Strong evidence suggests that the striatum plays an important role in the development of positive symptoms, as individuals with schizophrenia show an increased activation of this brain region at rest. One of the mechanisms underlying Avatar therapy may involve a reduction in aberrant salience. However, the neural processes involved in this therapeutic approach remain largely unexplored.

**Objectives:**

This study aims to investigate the brain mechanisms underlying Avatar therapy in treatment-resistant schizophrenia by examining spontaneous brain activity at rest using functional magnetic resonance imaging (fMRI).

**Methods:**

Fourteen participants with treatment-resistant schizophrenia participated in nine sessions of Avatar therapy. They underwent resting-state fMRI scans before and after the therapy. Voxel-wise analyses of fractional amplitude of low-frequency fluctuations (fALFF) were performed to examine differences between post- and pre-therapy in regional patterns of spontaneous brain activity.

**Results:**

Importantly, after the therapy compared to before the therapy, we found that participants with treatment-resistant schizophrenia had reduced fALFF in the right putamen. We also observed increased fALFF in the bilateral occipital, right inferior temporal, right angular gyrus, left medial temporal and left supramarginal gyri after the therapy.

**Conclusions:**

The putamen is a part of the striatum which is known to play a significant role in the emergence of psychosis. Mainly, our results suggest that Avatar therapy regulates putamen activity at rest, suggesting that the neural mechanisms underlying the therapy involve the alleviation of aberrant salience.

**Disclosure of Interest:**

None Declared

